# Current Imaging Modalities for assessing Ocular Blood Flow in Glaucoma

**DOI:** 10.5005/jp-journals-10008-1210

**Published:** 2016-10-29

**Authors:** Chirayu Mohindroo, Parul Ichhpujani, Suresh Kumar

**Affiliations:** 1 Intern, Department of Ophthalmology, Government Medical College and Hospital, Chandigarh, India; 2 Associate Professor, Department of Ophthalmology, Government Medical College and Hospital, Chandigarh, India; 3 Professor, Department of Ophthalmology, Government Medical College and Hospital, Chandigarh, India

**Keywords:** Color Doppler imaging, Glaucoma, Ocular perfusion pressure, Retrobulbar blood flow.

## Abstract

Glaucoma may be caused by an interplay of elevated intraocular pressure (IOP), vascular, genetic, anatomical, brain, and immune factors. The direct assessment of ocular hemodynam-ics offers promise for glaucoma detection, differentiation, and possibly new treatment modalities. All the methods currently in use to measure ocular blood flow have inherent limitations and measure different aspects of ocular blood flow. This review article attempts to provide detailed information on ocular perfu-sion pressure as well as an overview of the newly developed imaging technologies used to investigate ocular blood flow in glaucoma patients.

**How to cite this article:**

Mohindroo C, Ichhpujani P, Kumar S. Current Imaging Modalities for assessing Ocular Blood Flow in Glaucoma. J Curr Glaucoma Pract 2016;10(3):104-112.

## ANATOMICAL ASPECTS

Two different vascular beds, retina and choroid, nourish the posterior pole of the eye. They vary anatomically and physiologically, thus complicating the measurement of blood flow in the region. The long and short ciliary arteries supply the choroidal part. The anterior ciliary arteries mainly supply the large arteries in the outer portion of the choroid.^1^ These arteries branch into smaller vessels, which in turn feed the highly anastomosed choriocapillaris network lying at the inner border of the choroid, adjacent to the retinal pigment epithelium and retinal photoreceptors.

The retinal part is mainly nourished by the central retinal artery, which enters the retina with the optic nerve at the optic disk. The artery branches into radial arterioles and smaller vessels on the vitreal surface of the retina.^2^ Blood is returned through radial venules on the retinal surface that empty into the central retinal vein in the optic nerve. The total blood flow supplied to the choroid is much greater than that supplied to the retina. Many techniques have been used to provide details of the retinal circulation, but owing to the choroidal circulation, the precise details are compromised.

The fine branches from the circle of Zinn and the posterior ciliary arteries (PCAs) supply the optic nerve head (ONH) in the region of the lamina cribrosa. There is no contribution by the central retinal artery in this region. The prelaminar region is supplied by the peripapillary choroidal vessels, with some contribution from the vessels in the lamina cribrosa region. The blood supply to the ret-rolaminar portion of the optic nerve is through perforating vessels from the pia mater and also the short posterior ciliary arteries (SPCAs). The pial vessels obtain their supply either directly from the ophthalmic artery (OA) or indirectly from the recurrent branches from the SPCAs.^2^

## OCULAR BLOOD FLOW IN PRIMARY OPEN ANGLE GLAUCOMA AND NORMAL TENSION GLAUCOMA

Intraocular pressure (IOP) has been regarded as the only modifiable and treatable risk factor for glaucoma progression. Current research points toward vascular dysregu-lation as a significant cause for ganglion cell damage. Altered retinal and choroidal blood flow, decreased retrobulbar velocities, and higher retinal venous saturation are some factors that have been researched upon and hypothesized to have been involved in glaucoma progression.^3^ However, no study integrates all of the above or conclusively provides evidence for vascular dysfunction.

Primary open angle glaucoma (POAG) is characterized by open drainage angles on gonioscopy, glaucoma-tous optic disk damage and visual field loss, and an IOP less than 21 mm Hg, in the absence of secondary causes for optic disk damage.^4^ Various studies using different techniques found ocular hemodynamic deficits in patients with POAG compared with healthy individuals. However, the results need to be interpreted carefully. For example, there is enough evidence available regarding the abnormal flow velocity in retrobulbar vessels supplying the optic disk and nerve. However, a recent study by Zvia et al that focused on the blood supply of the central macular region reported that no abnormal velocity values were found between the glaucoma groups compared with healthy subjects. Hence, the parameters and the techniques need to be standardized for detecting vascular abnormalities in glaucoma patients. Our review focuses on the latter.

Similarly, normal tension glaucoma (NTG) is considered as a type of POAG, but IOP is equal to or below that of the threshold (21 mm Hg).^6^ Vascular insufficiency and decreased optic disk resistance are regarded as the major components, which cause damage in NTG. This makes the study of blood flow in NTG extremely essential. There have been a number of studies regarding the same, although with conflicting results. Briefly, Samsudin et al^7^ compared NTG patients with healthy subjects and reported that there was no difference in OA flow parameters between patients with NTG and controls. Bossuyt et al^8^ reported that vascular dysregulation in NTG was not affected by the structure and function of the microcirculation or macrocirculation, including arterial stiffness, total peripheral resistance, cardiac output, and both peripheral and central hemodynamics. However, the studies conducted by Plange et al,^9^ and Butt et al^10^ were contradictory where they found significant association between ocular blood flow parameters and NTG.

Because NTG is regarded as a variant of POAG, there have been studies comparing the two groups as well. One of the studies concluded that the vascular resistance of the OA could be associated with the development of visual field defects in NTG patients vis-a-vis patients with POAG.^2^ Another study evaluated the blood flow in patients with POAG and NTG and reported that the blood flows in the OA, central retinal artery, and PCAs^11^ were reduced in both forms of glaucoma, compared with controls.

A study worth mentioning in this section would be the Leuven Eye Study. This study created a database of over 600 patients over a 9-month period, comprising over five groups (healthy controls, POAG, NTG, ocular hypertension, and glaucoma suspects) and creating one of the largest databases in this field of study. The results of the study are beyond our scope of discussion. Briefly, they observed that glaucoma groups had lower retro-bulbar velocities and higher retinal venous saturation compared with the healthy group, which was in line with the current literature,^3^ essentially implying that vascular parameters could play a moderate role in the pathogenesis of glaucoma.

## TECHNIQUES AVAILABLE TO ASSESS OCULAR BLOOD FLOW

### Laser Speckle Flowgraphy

Principle

Laser speckle is an interference phenomenon that occurs when a coherent light, for example, laser light, is dispersed from a diffusing surface, i.e., retinal, choroi-dal vessels and the circulation of the ONH. This results in a rapidly varying pattern; this rate of variation can be utilized to determine the red blood cell velocity, which can be quantified to establish the retinal blood flow (RBF).^12^

Technique and Instrumentation

**Fig. 1:**
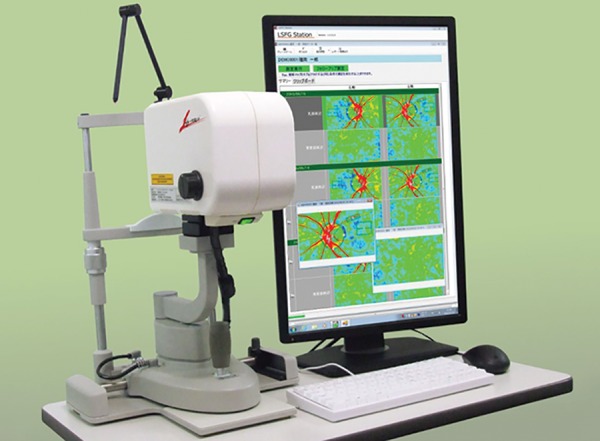
Laser speckle flowgraphy (LSFG-NAVI, Softcare Co., Ltd., Japan)

The instrumentation includes a fundus camera equipped with a diode laser (wavelength 808 nm), an image sensor, an infrared charge-coupled device (CCD) camera, and a high-resolution digital CCD camera. The diode laser and the image sensor are used for the laser speckle measurements. The infrared CCD camera observes the area of the fundus on which the laser beam is focused, and a high-resolution digital CCD camera is used to measure the retinal vessel diameter and record fundus photographs (Fig. 1). The scattered laser light is imaged on an image sensor corresponding to a field area of 1.06 × 1.06 mm or 0.72 × 0.72 mm; according to the magnification of the fundus camera, a speckle pattern is then seen on the fundus. The scanning speed of the image sensor is 512 scans/second. The structure of the pattern varies rapidly in accordance with the movement of blood cells in the tissue. The greater the blood cell velocity, the greater is the rate of variation. Each successive scan of the image sensor results in a different profile of the output signal intensity. The diode laser is used for measurements of circulation in the ONH, choroid, or iris.

Advantages

This technique is capable of measuring the relative blood flow velocity as the mean blur rate (MBR) by a noncontact and noninvasive method.^13^

Limitations

These laser-based techniques measure the flux, which depend on the scattering and optical absorption properties of the tissue. Due to differences in the tissue structure and composition in different eyes, the flux values cannot be compared directly. Furthermore, for a valid comparison between the flux values obtained at different times in the same eye, the tissue must be assumed to maintain the same scattering properties over time, which may not be the case. Additionally, the MBR offers only a relative blood velocity measurement, not an absolute one.

Literature Search

One of the preliminary studies conducted by Aizawa et al^14^ using laser speckle flowgraphy (LSFG) allowed them to quantify the microcirculation in the ONH examining glaucoma patients. Their study indicated how LSFG could be a potential tool for the assessment of microcirculation in glaucoma patients. Yokoyama et al^15^ compared 60 eyes with myopic glaucomatous discs with 24 eyes with no ocular diseases. Their results indicated that the average MBR for the entire optic disk was significantly lower in the glaucoma group than that in the control group, thus validating the rationale behind using LSFG in glaucoma patients. Most recently, a study done on a larger scale has concluded that LSFG was a good, noninvasive technique for the identification of glaucoma and the classification of its severity in eyes with myopic optic disks.^16^

What's New?

A recent advancement overcoming the aforementioned hurdles is a newer version of LSFG's accompanying software, LSFG Analyzer (Version 3.1.6; Softcare Co., Ltd., Fukutsu, Japan), which provides a relative flow volume. Subtraction of the background choroidal blood flow from the overall blood flow value of a region of interest centered on a retinal vessel reflects the retinal flow velocity and vascular diameter.^13^

### Color Doppler Imaging

Principle

Color Doppler imaging (CDI) is an ultrasound technique using a combination of B-scan (gray-scale imaging of tissue structure) along with colored representation of blood flow utilizing Doppler-shifted frequencies and pulsed Doppler measurement of blood flow velocities. It is used for measuring retrobulbar velocities in the eye.^17^

Technique and Instrumentation

Color Doppler imaging is performed using a phased array transducer, with an ultrasound frequency of 6.5 MHz in the pulsed Doppler mode. The examination is conducted with the patient in a supine position and the head elevated at about 30° angle. The ocular examination usually takes about 30 to 40 minutes. A transducer is carefully set on the closed eyelids, using an acoustic coupling material, such as a carbomeric gel, without exerting pressure on the bulb. In the first instance, a B-scan of the optic nerve is obtained, which provides the most useful landmark for the identification of the retrobulbar vessels. The OA, which is a branch of the internal carotid artery, is situated above or below the optic nerve in the posterior orbit and passes forward into the nasal orbit. After crossing the optic nerve, the OA can be traced through the flow toward the applicator and by the typical pulsatility approximately 15 mm posterior to the globe in every individual. The SPCAs, usually about 6 to 12 in number, arise from the OA or its branches. They begin as trunks approximately 10 to 20 mm behind the globe, before they form multiple branches surrounding the optic nerve in its retrobulbar portion. They pierce the sclera around the optic nerve and supply the choroid and ciliary processes. Because of the high variability of the course, the first point behind the globe where they can be measured to show the characteristic Doppler spectra is the point to get the best reproducibility. The two long PCAs can be localized more distal from the optic nerve and can be measured directly behind the sclera before their entry into the sclera. They run forward, along either side of the eyeball, between the sclera and the choroids to supply the anterior part of the eye with blood. It is important to have a correct angle between the transducer and the orientation of the vessel. Gain and threshold must be adjusted individually for each examination until noise disappears.^17^

Advantages

It is a great tool to assess the large ophthalmic vessels, such as the OA, the central retinal artery, and the SPCAs. The peak systolic velocity (PSV) and the end diastolic velocity (EDV) represent the fastest velocities at the systole and the diastole respectively. Further, the mean velocity can be calculated and the resistive index (RI) can be determined.^18^ However, the RI [(PSV - EDV)/PSV] does not accurately correlate with the resistance offered by the ophthalmic vessels.

Limitations

Color Doppler imaging is an excellent way to assess the large arteries, but quantitative information of the vessel diameter cannot be obtained. Therefore, the total blood flow cannot be calculated with this technique.^1^ Moreover, owing to the anatomical differences among individuals, in density and organization of the vessels, along with the fact that CDI is highly dependent on probe placement, the technique is yet to be standardized.^17^

Literature Search

Color Doppler imaging variables are said to strongly correlate with visual field loss in POAG. One of the prospective studies done by Galassi et al^19^ shows the effect of blood flow velocity in terms of EDV and RI on the progression of visual field damage in patients with POAG over a period of 7 years, thus highlighting the importance of CDI for predicting disease progression in glaucoma. Enough literature is available on abnormal erythrocyte velocity in the OA, central retinal artery, and SPCA with POAG. Besides disease progression in POAG, several types of glaucoma have also been compared. A study by Marjanovic et al^20^ compared 52 eyes from 52 patients with POAG and 25 eyes from 25 acute angle closure (ACG) patients. They reported that the RI was significantly higher in both the OA and SPCA in the POAG patients compared with that in ACG patients. This indicated the importance of CDI specifically for POAG patients. Reduction in the blood flow velocities and increase in the RIs were also detected in most retrobulbar vessels that were found in patients with NTG.^18^ Hence, we can conclude that CDI could be used as an excellent tool for assessing the blood flow in various types of glaucoma and assessing the disease progression.

### Doppler Fourier Domain-Optical Coherence Tomography

Principle

The Fourier domain-optical coherence tomography (FD-OCT) is a noncontact and noninvasive imaging technique that utilizes a micrometer-scale resolution with a millimeter image penetration depth, based on low-coherence interferometry. This technique perceives the intensity of light, scattered back from the moving erythrocytes within the vessels of the ocular tissue, inducing a Doppler frequency shift that represents the velocity component parallel to the axis of the probing beam.^21^ This frequency shift introduces a phase shift in the spectral interference pattern, i.e., captured by a line camera. The spectral information is converted into complex axial scans containing both amplitude and phase using the fast Fourier transform. The phase differences between sequential axial scans at each pixel are calculated to determine the Doppler shift.

Technique and Instrumentation

Doppler FD-OCT generates high-resolution cross-sectional images of the retina. This instrument utilizes a laser light source of 841 nm with a bandwidth of 49 nm and an incident power of 500 μW on the cornea. These parameters result in an axial resolution of 5.4 μm in tissue. The system transverse resolution is 20 μm, as determined by the maximum aperture of the eye. Unlike morphological FD-OCT systems that produce just structural images, the prototype Doppler FD-OCT analyzes the Doppler phase shift between two consecutive A-scans. The light reflected from moving particles undergoes a Doppler phase shift.

Flow velocity is determined by

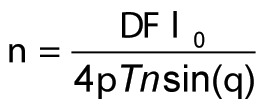



where *v* is the flow velocity in an OCT voxel

ΔΦ is the Doppler phase shift (ΔΦ = Φ1 - Φ2), where Φ1 and Φ2 are the phases of voxels in the same position in consecutive OCT axial scans

λ_0_ is the source center wavelength

*n* is the refractive index of the medium

*T* is the time interval between consecutive scans

*θ* is the Doppler angle defined by the OCT beam axis relative to the line perpendicular to blood vessel flow axis.

**Fig. 2:**
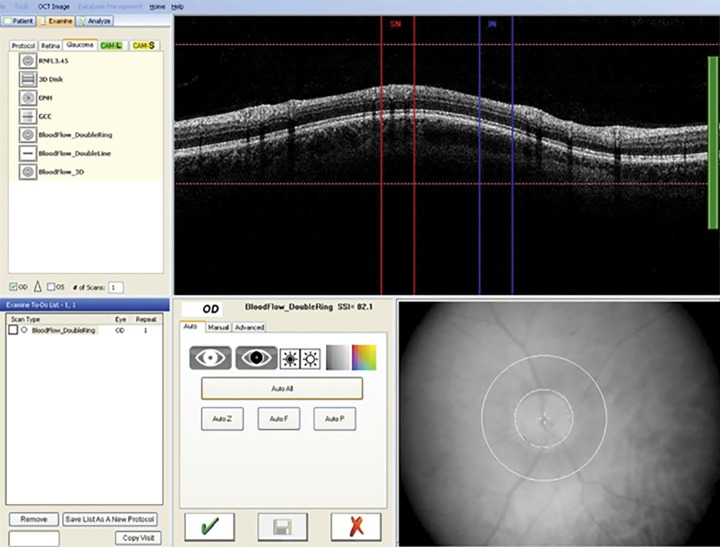
Double circular Doppler scan (two concentric rings around the optic nerve)

The maximum detectable Doppler phase shift is determined by the acquisition speed of the CCD camera. Given this setup, the maximum measurable velocity in the retinal vessels has been found to be 2.8 mm/second. Phase detection caused by the RBF has been incorporated into the prototype system by creating two circular scans centered on the ONH. The RBF protocol consists of a double circular Doppler scan comprising two concentric rings 3.4 and 3.75 mm in diameter centered on the ONH (Fig. 2). The double circular FD-OCT beam passes through the pupil nasally with two sets of scans (inferior and superior). The circular scan is displayed as the sinusoidal variation in retinal height/morphology. The incident angle is estimated by the vessel center depth difference within the two consecutive circular OCT scans from the Doppler FD-OCT images. The measured Doppler phase shift, the incident angle calculation, and the vessel area are used to compute the absolute red blood cell velocity and RBF.^21^

Advantages

This is a noncontact, noninvasive technique capable of truly measuring the total volumetric RBF. Moreover, it is not limited to only large vessels or a single measurement site.^1^ Another advantage is the excellent quality of resolution used to detect the retinal vasculature.

Literature Search

In the studies conducted by Tayyari et al,^21^ the values generated for the RBF were comparable with laser Doppler velocimetry (LDV), which is regarded as one of the most established methods of determining RBF. Wang et al^22^ performed the first study using four-dimensional (4D)-OCT in 19 glaucoma patients. They reported significantly lower arterial and venous velocities compared with normal subjects; the arterial and venous cross-sectional areas were the same as normal. Moreover, the decrease in velocities could also be associated with disease progression. This study was essential as it established the fact that 4D-OCT could be a useful tool in the assessment of severity and diagnosis of glaucoma. Another study done on a larger scale yielded similar results.^23^ A strong correlation between reduced RBF and visual field loss in glaucoma was established with this study.

Limitations

This technique is mainly limited by eye motion, tear film, and the anatomy of the vessels. For example, tear breakup can alter the laser intensity projected onto the retina and may result in displacement of the laser from the center of the vessel due to optical blurring effects. Also dietary factors, such as caffeine or consuming red meat could alter the blood flow resulting in false values. Then the fundamental error components also need to be accounted for, such as Doppler angle error caused by motion error, vessel boundary segmentation error, Doppler phase error due to phase wrapping, residual bulk motion error (after compensation), and system phase noise.^21^ Then the objects that are close to perpendicular to the incident OCT beam will result in a relatively weak Doppler signal because the cosine of 90° is zero. Errors in the measurement of the vessel lumen area can greatly influence the estimation of flow, and also the impact of eye movements will introduce further error into the estimation of vessel lumen area.

What is New?

Dai et al^24^ have developed a novel dual-beam OCT technique that can measure the Doppler angle and the absolute blood flow velocity of retinal vessels by using two simultaneously acquired B-scan images covering the same vessel with a predefined distance. Because the two OCT images are acquired simultaneously, the measurement overcomes difficulties, such as eye motion and motion artifacts.

### Laser Doppler Velocimetry

Principle

Bidirectional LDV is a technique based on the Poiseuille principle and the Doppler effect. The instrument detects the change in frequency due to reflected laser light by a moving particle. The change in frequency is proportional to the velocity of the moving particle. For each vessel, the maximum velocity is calculated (which is said to be in the center of the vessel), and the laser light scattered from stationary tissue is taken as the reference beam from which a relative change in RBF speeds can be mea-sured.^25^ By utilizing two photomultipliers separated by a known angle, the frequency shifts are subtracted to remove the uncertainty of scattering angles. This is how the centerline blood velocity (mm/second) and vessel diameter (m) are determined and further total flow is calculated in L/min.^26^

Technique and Instrumentation

A red diode measurement laser (675 nm, 80 m, 50 m oval) is used to measure up to a maximum velocity of 120 mm/ second. The measurement window of 2 seconds gives continuous velocity readings (every 0.02 seconds) and plots a velocity-time curve. Calibration of the laser blood flowmeter (LBF) to measure the blood velocity and vessel diameter is done during assembly. Once installed, periodic adjustments of laser power and detector sensitivity and daily optical system checks are undertaken to maintain the calibration of the instrument. The LBF also uses a green diode vessel tracking laser system (543 nm), i.e., used to stabilize, and measure the diameter of, the vessel of interest.^27^ The vessel tracking system allows a graph of eye position to be superimposed on the velocity-time curve to aid in artifact rejection. Multiple diameter readings are acquired during the first and final 60 ms of the 2-second velocity measurement window every 0.04 ms. Two sequential bidirectional readings (i.e., path 1 and 2) are taken to ensure consistency and averaged to give one reading. In combination with the average velocity over a pulse cycle and the diameter, the flow through the vessel can be calculated as *FV_max_* S/2, where S is the cross-sectional area of the vessel at the measurement site.

Advantages

The technique is noninvasive and gives consistent and repeatable measurements of blood flow within retinal arterioles. It is capable of calculating the blood velocity, cross-sectional area, vessel diameter, and hence the total blood flow. It is one of the few devices that can measure retinal volumetric blood flow in absolute units.

Literature Search

Laser Doppler velocimetry is a modality that has been extensively researched; yet, owing to the complexity of the instrument, the clinical use has not been established. Some efforts have been made to simplify the technique. Laser Doppler velocimetry has been utilized to study the effect of a variety of antiglaucoma medications and experimental drugs on ocular blood flow. Timolol maleate,^28^ acetazolamide,^29^ and carteolol^30^ are few drugs whose effects have been studied with LDV. More recently, the effect of moxaverine, a phosphodiesterase inhibitor, was determined in 20 POAG subjects.^31^

Limitations

The major factors that affect the measurements are eye motion and centerline displacement. Minor factors, such as tear film breakup, inadequate dilation, upper lid obstruction, and media opacities can also cause blurring of images generated.^26^ Also LDV cannot be used to measure the circulation in ONH.^1^

### Confocal Scanning Laser Doppler Flowmetry

Principle

This technique utilizes an infrared laser to scan the retina combining the laser Doppler flowmetry with confocal scanning laser tomography. The frequency and amplitude of Doppler shifts in the reflected light allow for determination of blood velocity and blood volume respectively. This information is used to compute the total blood flow and to create a physical map of flow values contained in the retina.^32^

Technique and Instrumentation

A prototype is Heidelberg retinal flowmeter (HRF). Heidelberg retinal flowmeter is a confocal scanning laser Doppler scanning that images a 2560 × 2560 × 400 μm volume of the retina or ONH with a 780 nm scanning infrared laser. Two green lines on the operator screen mark the boundaries of the 2560 × 640 × 400 μm volume of tissue from which flow data will be derived. After the laser beam is centered on the area of interest, focus is adjusted to produce maximal brightness within that area. With laser position and focus optimized, sensitivity is set so that the brightest pixels within the area of interest are light yellow in color. White pixels are avoided. When acceptable alignment, focus, and brightness are achieved, the operator initiates a process that, within 2.048 seconds, scans each of 64 lines 128 times. Lines are scanned several times to yield an acceptable signal-to-noise ratio. After the scan is completed, the operator initiates a fast Fourier transform to extract the individual frequency components of the reflected light. For each point of the scan (256 columns × 64 rows of pixels), a frequency power spectrum is calculated. On the x-axis of the spectrum, each frequency location represents the blood velocity, and the height of the spectrum at that frequency represents the number of blood cells required to produce that intensity. Integrating the spectrum yields the total blood flow. For analysis of selected 100 × 100 × 400 μm of tissue, a sample box is placed on an area free from motion artifacts and major vessels are selected. These small boxes are in fact the default sample size used in analyses by the instrument. Printed HRF reports, which show the position of the sample window relative to the microvasculature of the ONH and peripapillary retina, are used to guide subsequent measurements to a site within 10 μm of the baseline measurement relative to the vasculature. When flow histograms are generated from the entire image, velocity, volume, and flow are computed for each 10 × 10 μm pixel within the 2560 × 640 × 400 μm image; after elimination of pixels that contain major vessels, are poorly focused, or are improperly illuminated, the remaining values are computer sorted based on the flow. The mean flow for the entire array is computed, the number of zero flow pixels (“avascularity”) is determined as a percentage of total pixels, and the flow in the 25th, 50th, 75th, 90th, 95th, 99th, and 100th percentile pixels is determined.^32^

Advantages

Confocal scanning laser Doppler flowmetry (CSLDF) is one of the most widely utilized techniques to study blood flow in glaucoma. It generates two-dimensional maps of retinal and ONH capillary perfusion bed. More importantly, it provides a “full-field” analysis, which can be helpful in gradation of the severity of the disease.^33^

Literature Search

Confocal scanning laser Doppler flowmetry has been successfully used in studying ocular perfusion in glaucoma patients. Various studies examining the overall retina and some specific areas, such as the ONH have been done to correlate the blood flow with either grading the severity of the disease or diagnosing the disease itself. Logan et al^34^ successfully demonstrated the decreased blood flow in glaucoma eyes using this very technique. Further studies have been done comparing POAG, NTG, and diseases, such as exfoliation syndrome justifying the use of this technology. Recently, a study comparing the African descent population with European descent population over a period of 3 years using CSLDF has found that individuals of African descent may have a stronger vascular component to their glaucoma pathophysiology than patients of European descent.^35^

Limitations

Confocal scanning laser Doppler flowmetry is highly sensitive to illumination changes and eye movement, and it measures the blood flow within only a relatively small velocity range. Furthermore, small changes in the sample window placement can yield large differences in flow measurement, leading to relatively limited repro-ducibility. Application of improved and sophisticated software may yield better results.^32,33^

### Retinal Functional Imager

Principle

The retinal functional imager (RFI) is a noninvasive diagnostic approach for measuring the blood flow velocity of medium-sized vessels in the retina. The principle underlying its action is that the device identifies the motion of red blood cells in retinal vessels by comparing several images of the retina taken under green light within a very short time interval. Software analysis calculates the distance traveled by red blood cells in a certain known time, giving a measure of their velocity.^36^

Technique and Instrumentation

**Fig. 3:**
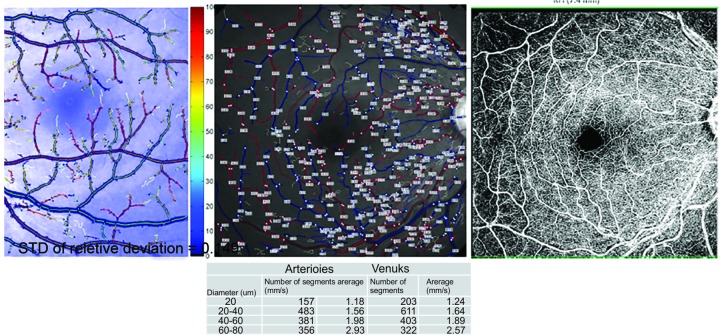
Wide-field noninvasive oximetry, velocity, flow, and angiography with RFI (Optical Imaging Ltd.)

The prototype RFI system (RFI 3005, Optical Imaging Ltd., Rehovot, Israel) is based on a standard fundus camera extended by a customized stroboscopic flash lamp system and a digital camera. The blood flow velocity is measured by quantifying the movement of hemoglobin-containing erythrocytes, as hemoglobin is a natural high-contrast chromophore that marks the flow of blood and thus facilitates the calculation of the blood flow velocity. A green (“red-free”) interference filter is used for illumination, with transmission centered at 548 nm at a bandwidth of 17 nm. The fundus camera employs a 60 Hz 1024 1024 pixel digital imaging system, delivering eight consecutive flashes typically at intervals of 17.5 ms to generate eight consecutive fundus images. A patient's heartbeats are monitored by a probe attached to the fingertip or the earlobe of the patient in order to synchronize image acquisition at a given period of the cardiac cycle and thus neutralize the effects of pulsation of the arterial blood flow velocity (Fig. 3).^37,38^

Advantages

The RFI device, introduced less than a decade ago, offers noninvasive imaging capabilities that are very attractive for the clinical evaluation of the RBF velocity in the arte-rioles and venules of the retina. The device also provides a means to image the capillary perfusion map with the foveal avascular zone, along with the option to measure vessel oxygenation and even metabolic mapping of the retinal tissue.^36-39^

Limitations

Retinal functional imager provides only flow velocity data and not flow volume information due to inaccuracy of vessel width measurement. In addition, the vessel segment length and field of view can also alter the results.^36^

Literature Search

A study was conducted to measure the blood flow velocity in 59 eyes of 46 patients with POAG and 28 eyes of 23 patients with glaucomatous optic neuropathy using RFI. The results indicated changes in the RBF velocity only in the preperimetric state, but not in perimetric glaucoma.^39^ Although only the central macular region was examined, the authors strongly suggested the use of RFI in glaucoma for detecting glaucoma in early stages.

There are still many ophthalmologists who fail to diagnose the vascular problems of their glaucoma patients and do not consider them when treating patients. With the emergence of new risk factors, such as disturbed ocular blood flow or vascular dysregulation, the newer investigative modalities discussed in this review may help to target these risk factors with more specific treatment strategies.
